# Public opinion and medical cannabis policies: examining the role of underlying beliefs and national medical cannabis policies

**DOI:** 10.1186/s12954-015-0082-x

**Published:** 2015-10-14

**Authors:** Sharon R. Sznitman, Anne Line Bretteville-Jensen

**Affiliations:** School of Public Health, University of Haifa, Eshkol Tower, room 705, Mt. Carmel, 3190501 Haifa Israel; Norwegian Institute for Alcohol and Drug Research, Post Box 565, Sentrum, 0105 Oslo Norway

**Keywords:** Medical cannabis policies, Legalization, Public opinion, Cross-national comparison

## Abstract

**Background:**

Debate about medical cannabis legalization are typically informed by three beliefs: (1) cannabis has medical effects, (2) medical cannabis is addictive and (3) medical cannabis legalization leads to increased used of cannabis for recreational purposes (spillover effects). We examined how strongly these beliefs are associated with public support for medical cannabis legalization and whether this association differs across divergent medical cannabis policy regimes.

**Methods:**

Robust regression analysis was used to analyse data derived from two nationally representative samples of adults participating in comparable cross-sectional online surveys in one country where medical cannabis smoking is illegal (Norway, *n* = 2175, 51 % male) and in one country where medical cannabis smoking is legal (Israel, *n* = 648, 49 % male).

**Results:**

The belief that cannabis has medical benefits was more strongly related to support for medical cannabis legalization than were beliefs about addiction and spillover effects. While the support for medical cannabis legalization was stronger in Israel than in Norway (78 vs. 51 %, *p* < 0.01), the belief variables had, in general, more impact on the policy stand in Norway.

**Conclusion:**

The belief that cannabis has medical benefits is particularly salient for support for medical cannabis legalization. It is possible that the recent surge in evidence supporting the medical benefits of cannabis will increase the belief about medical benefits of cannabis in the general population which may in turn increase public support for medical cannabis legalization. Results also suggest that once medical cannabis is legalized, factors beyond cannabis-specific beliefs will increasingly influence medical cannabis legalization support. These conclusions are, however, only suggestive as the current study is based on cross-sectional data. Hopefully, future research will be able to capitalize on changes in medical cannabis policies and conduct longitudinal studies that enable an examination of the causal relation between public opinion and medical cannabis policy changes.

## Introduction

Medical cannabis policies are currently undergoing rapid changes, with increasingly more jurisdictions around the world legalizing medical cannabis for certain groups of patients. These include 23 states in the USA [1] and countries like Israel, Canada and the Netherlands [2]. Other nations are currently considering the same policy change, e.g. New Zealand and Australia [1, 3]. At the same time, there are many more countries where medical cannabis legalization is absent from the current policy agenda and public debates.

Although very little peer-reviewed scientific literature exists on public opinion towards medical cannabis policy, it has been noted that public opinion has played an important role in affecting medical cannabis policy formation, modification and support [1]. To understand the dynamics of medical cannabis policies and to begin to create a framework for forecasting future developments in this policy area, there is a need to examine public support for medical cannabis legalization. Furthermore, as public support can influence and at the same time be influenced by national policies, it is imperative to study public support across different medical cannabis policy regimes, as this enables an investigation of whether differences in public opinion reflect variation in medical cannabis policies. In this paper, we examine the extent to which beliefs, intrinsic to claims frequently made in the medical cannabis debate, relate to support for medical cannabis legalization and whether the relationships differ across divergent medical cannabis policy regimes.

There are three common beliefs that are frequently deliberated in public and policy debates of medical cannabis legalization. The first belief is about the medical benefits on cannabis, and many question the therapeutic value of cannabis in clinical practice [1]. Proponents of medical cannabis highlight the continuing emergence of evidence that demonstrates the therapeutic value of cannabis in treating a variety of disease-related problems [4, 5].

Secondly, medical cannabis legalization debates have revolved around topics of addiction and abuse. Official policies mandated by the 1961 UN Convention highlight the abuse potential of cannabis, and there is a vast research literature that identifies harmful effects of cannabis use [6]. While opponents of medical cannabis have highlighted the abuse potential, proponents have noted that cannabis is far less addictive and harmful than other prescription medicines [7, 8].

Thirdly, spillover effects are often discussed in debates about cannabis legalization, including the concern that medical cannabis legalization may lead to increased recreational cannabis use. There are various ways in which this is thought to occur. It has, for instance, been argued that medical cannabis legalization leads to diversion of medical cannabis to the black market [9]. Others have noted that medical cannabis legalization leads to increased recreational cannabis use because it sends “the wrong messages to the public” that cannabis for recreational purposes is acceptable and that there is little risk to the user and society [9–11]. This is closely connected with the fact that medical cannabis is typically smoked, which raises the concern that medical cannabis legalization blurs the boundaries between cannabis for therapeutic purposes and cannabis for recreational purposes [12].

### Study objectives

The aims of our study were twofold. Firstly, we examined the extent to which beliefs underlying the medical cannabis debate relate to support for medical cannabis legalization. On the one hand, one may expect that believing that cannabis has medical benefits will be associated with support for medical cannabis legalization and that beliefs in the negative effects of medical cannabis (e.g. addiction and spillover effects) will be associated with opposition to medical cannabis legalization. However, the converse is also theoretically plausible. It is possible that cannabis is generally accepted as a medicine, which in turn goes hand in hand with the acceptance that a certain level of unintended negative effects (e.g. addiction and spillover effects) is inevitable. Seen from this perspective, it is possible that addiction and spillover beliefs are positively rather than negatively related to medical cannabis legalization.

Secondly, our study aimed to examine if and how support for medical cannabis legalization differs across divergent medical cannabis policy regimes. Ideally, we would have liked to compare data pre- and post-policy changes across different legal jurisdictions within a country. However, such “natural experiment” data are rare in the social sciences and are not available here. Thus, we chose the next best option which was to compare individual level data collected in two countries (Israel and Norway) that have many similarities but which differ with regard to medical cannabis policies.

## Methods

We used a cross-national data set to compare public opinion towards medical cannabis legalization between Norway and Israel. Respondents read and agreed to an informed consent form before filling in the survey. In Norway, a nationally representative sample (*n* = 2175) of the adult (aged 18+) population was recruited for a Norwegian language online survey. In Israel, a nationally representative sample (*n* = 648) of the adult (aged 18+) Jewish population was recruited for a Hebrew language online survey. The two national surveys included identical questions about cannabis policy attitudes and beliefs, cannabis use, demographics and other background factors. IRB approval was granted by the Social Welfare and Health Sciences Department at the University of Haifa (nr. 289/13).

### Dependent variable

Respondents were asked to assess the extent to which they agreed that cannabis use should be legal for patients if a physician recommends it. Response categories were presented on a Likert scale indicating the range from strongly disagree (=1) to strongly agree (=5). There was also a “don’t know” category which was combined with the Likert scale midpoint “neither agree nor disagree” category.

### Covariates

Previous research has found that gender, education, age, alcohol and drug use experiences relate to drug policy support [13–15]. Therefore, the following control variables were coded for inclusion in the analysis: gender (0 = female, 1 = male), dummy age categories (18–29, 30–44, 45–59, 60+), dummy variables for highest completed education level (primary, secondary and higher education), last year cannabis use (0 = no, 1 = yes) and last month alcohol use (0 = no, 1 = yes).

### Belief variables

The three common beliefs underlying the medical cannabis debate were explored. The beliefs that (1) cannabis has medical benefits and that (2) medical cannabis is addictive were recorded separately on a 5-point scale ranging from 1 = strongly disagree to 5 = strongly agree. There was also a “don’t know” category which was combined with the Likert scale midpoint “neither agree nor disagree” category. To explore the third belief item, we asked respondents how likely they thought that medical cannabis legalization led to increased cannabis use for non-medical purposes. Response categories ranged from very unlikely (=1) to very likely (=4).

### National medical cannabis policy variable

To examine public opinion as a function of medical cannabis policy regime, a variable indicating whether respondents were from Norway (=0) or Israel (=1) was coded.

Israel and Norway share important population and geographic characteristics including relatively small population sizes (five million in Norway and eight million in Israel), and both are not members of the European Union despite being in close proximity to Europe. Furthermore, both countries define cannabis as a schedule 1 drug of abuse, and both enforce criminal prosecution against personal use and trafficking of cannabis. By international standards, Israel and Norway have relatively low cannabis prevalence rates [16–18]. A national representative survey in Israel showed that 8.9 % of the adult population (18–40 years) reported having used cannabis in the previous year [19]. In Norway, the corresponding last year cannabis prevalence (16–34 years) was 7.0 % [20].

In contrast to these similarities, Norway and Israel represent divergent medical cannabis policy regimes which enable an investigation of the extent to which medical cannabis policy support relates to actual national medical cannabis policies. Israel has been running a medical cannabis program since the late 1990s [21] in which the use of cannabis is allowed after specialist physicians’ recommendation and medical cannabis licence approval. Individual patients obtain medical cannabis licences which specify monthly dosage and medical cannabis supplier. Medical cannabis suppliers produce high-grade medical cannabis that is most commonly administered by smoking or vaporizing. In recent years, the rate of medical cannabis licences granted in Israel has grown significantly—from just a few hundred in 2007 to an estimated 22,000 in 2015 [22]. Medical cannabis has also been subject to much public and media debate, and newspaper reports on medical cannabis have tended to describe cannabis as a prescription drug rather than a recreational drug [23].

In Norway, medical cannabis in smoked form is not allowed. In 2012, prescription of Sativex®, a cannabis extract which is administered orally with an oromucosal spray, was approved. Potentially, due to the fact that Sativex® is administered by spray and not smoking, the prescription of Sativex® has not generated much public debate or media attention in Norway. The use so far is limited; by the end of 2013, there were 402 registered Sativex® patients and the number decreased to 365 patients in 2014.

### Analytical strategy

For descriptive statistics, proportional tests were used to determine national sample differences on key variables. Spearman correlations were used to examine the bivariate relation between public support for medical cannabis legalization and the beliefs commonly underlying the medical cannabis debate. Public support for medical cannabis legalization was further examined in multivariate linear regression models. To account for heterogeneity and lack of normality, Huber–White sandwich estimators were used to calculate robust standard errors [24]. In the first step of the multivariate regression analysis, we entered only the covariates and the country variable to examine country differences after control for background variables. Thereafter, we entered the belief variables to examine their relation to support for medical cannabis legalization net of background variables. Lastly, in order to examine the extent to which underlying beliefs are differently associated with support for medical cannabis policy in Israel and in Norway, interaction terms between belief variables and nation were entered one at a time. Marginal effects were calculated and presented graphically. In order to test the robustness of results, sensitivity analysis without cannabis users was conducted. Stata [25] was used for all analyses.

## Results

Table 1 presents unadjusted statistics and shows that there was more support for medical cannabis legalization in Israel than in Norway (78 % of Israeli respondents strongly agreed/agreed that medical cannabis should be legal [mean = 4.1] vs. 53 % of Norwegians [mean = 3.3], *p* < 0.001). The national sample distributions across gender and the older age groups (45+) were similar. However, there were slightly more respondents in the youngest age group in the Norwegian sample, whereas there were more respondents in the second youngest age group in the Israeli sample. Norwegians were more likely to have secondary education than Israelis. Norwegians were slightly more likely to report alcohol use in the previous month (74 vs. 69 %, *p* < 0.05), whereas Israelis were more likely than Norwegians to report cannabis use in the previous year (13 vs. 5 %, *p* < 0.001). Compared to Norwegians, Israelis were more likely to believe that cannabis has medical benefits (67 vs. 29 %, *p* < 0.001) and less likely to believe that medical cannabis is addictive (34 vs. 55 %, *p* < 0.001). An equal and high proportion of respondents in both countries endorsed the belief that medical cannabis legalization will increase recreational non-medical use (60 % in both countries, *p* > 0.05).

**Table 1:** Distribution of background and independent and dependent variables in Norway and Israel

	Norway (*n* = 2175)	Israel (*n* = 648)	*p* value for country difference
Strongly agree/agree that medical cannabis should be legal, % (*n*)	53.2 (1158)	78.4 (508)	
[Mean (SD)]	[3.3 (0.03)]	[4.1 (0.04)]	<0.001
Male, % (*n*)	50.8 (1104)	49.1 (318)	0.342
Age categories			
18–29, % (*n*)	26.4 (576)	22.1 (143)	0.003
30–44, % (*n*)	23.9 (519)	28.7 (186)	0.010
45–59, % (*n*)	30.0 (652)	28.9 (187)	0.795
60+, % (*n*)	19.7 (429)	20.4 (132)	0.416
Education			
Primary, % (*n*)	7.5 (164)	28.1 (182)	<0.001
Secondary, % (*n*)	62.2 (1353)	24.3 (157)	<0.001
Higher education, % (*n*)	30.2 (658)	47.6 (308)	<0.001
Behaviour			
Last month alcohol use, % (*n*)	74.3 (1616)	69.1 (448)	0.010
Last year cannabis use, % (*n*)	5.3 (115)	13.0 (84)	<0.001
Beliefs			
Strongly agree/agree that cannabis has medical benefits, % (*n*)	29.2 (635)	66.7 (432)	<0.001
Strongly agree/agree that medical cannabis is addictive, % (*n*)	54.5 (1186)	34.3 (222)	<0.001
Very likely/likely that medical cannabis legalization leads to increased cannabis use, % (*n*)	60.4 (1313)	60.3 (391)	0.953

*p* values are based on two-sample tests of proportions

Correlations between beliefs and support for medical cannabis legalization are presented separately for Israel and Norway in Table 2 (upper and lower part of the table). Support for medical cannabis legalization was most strongly and positively related with the belief that cannabis has medical benefits in both countries. However, while the correlation was strong in the Norwegian data (*r*_*s*_ = .71, *p* < 0.001), it was relatively weak in the Israeli data (*r*_*s*_ = .26, *p* < 0.001). A stronger belief that medical cannabis was addictive had significantly negative (albeit weak) association with support for medical cannabis legalization support in Norway (*r*_*s*_ = −.22, *p* < 0.001) and Israel (*r*_*s*_ = −.11, *p* < 0.001). The spillover belief was negatively related to legalization support in Norway (*r*_*s*_ = −.39, *p* < 0.001) while the relation was not significant in the Israeli sample (*r*_*s*_ = −.06, *p* > 0.05). Results presented in Table 2 also show that in both countries, the belief items are significantly but not strongly associated with each other.

**Table 2:** Spearman correlations in the Norwegian sample (lower half, *n* = 2175) and the Israeli sample (upper half, *n* = 648)

	Support for medical cannabis legalization	Cannabis has medical benefits	Medical cannabis is addictive	Spillover
Support for medical cannabis legalization	1.00	0.26**	−0.11**	−0.06
Cannabis has medical benefits	0.71***	1.00	−0.34***	0.27***
Medical cannabis is addictive	−0.22***	−0.20***	1.00	−0.35***
Spillover	−0.39***	0.32***	−0.33***	1.00

**p < 0.01; ***p < 0.001

The multivariate regression results presented in model 1 in Table 3 show that education and age were not independently associated with support for medical cannabis legalization (*p* > 0.05). Males (coeff. = 0.143, *p* < 0.01) and those who reported for recent use of alcohol (coeff. = 0.186, *p* < 0.01) and cannabis use (coeff. = 0.892, *p* < 0.001) were relatively supportive of medical cannabis legalization. Furthermore, the multivariate results confirm that Israelis are more likely to support medical cannabis legalization than their Norwegian counterparts (coeff. = 0.712, *p* < 0.001).

**Table 3:** Regression models predicting support for medical cannabis legalization (*n* = 2803)

Predictors	Model 1			Model 2			Model 3		
	Coeff.	Robust S.E.	*p* value	Coeff.	Robust S.E.	*p* value	Coeff.	Robust S.E.	*p* value
Demographics									
Male	0.143	0.054	0.007	0.114	0.041	0.005			
Age (referent: age category 18–29)									
Age 30–44	0.100	0.075	0.185	−0.036	0.057	0.529			
Age 45–59	0.133	0.073	0.068	0.038	0.055	0.496			
Age 60+	−0.152	0.081	0.062	−0.065	0.064	0.303			
Education (referent: primary)									
Secondary	−0.002	0.087	0.979	−0.132	0.074	0.077			
Higher education	0.135	0.086	0.118	0.029	0.076	0.702			
Behaviour									
Last month alcohol use	0.186	0.062	0.003	0.144	0.046	0.002			
Last year cannabis use	0.892	0.081	<0.001	0.087	0.072	0.226			
National policies									
Israel	0.712	0.061	<0.001	0.054	0.061	0.375			
Beliefs									
Cannabis has medical benefits				0.732	0.018	<0.001			
Medical cannabis is addictive				0.016	0.019	0.399			
Medical cannabis legalization leads to increased cannabis use				−0.236	0.033	<0.001			
Interaction terms									
Israel, cannabis has medical benefits							−0.625	0.052	<0.001
Israel, medical cannabis is addictive							0.235	0.049	<0.001
Israel, medical cannabis legalization leads to increased cannabis use							0.553	0.071	<0.001

*Coeff.* coefficient, *S.E.* standard errors

In model 2, Table 3, the three belief variables were added to the regression equation. Results show that a stronger belief that cannabis has medical benefits is associated with stronger support for medical cannabis legalization (coeff. = 0.732, *p* < 0.001), whereas a stronger belief that medical cannabis legalization will lead to increased non-medical cannabis use is associated with less support for medical cannabis legalization (coeff. = −0.236, *p* < 0.001). Believing that medical cannabis is addictive was not independently associated with medical cannabis legalization support (*p* > 0.05).

The multivariate regression model differed from the previous model in that it showed that after inclusion of belief variables, there were no national differences in support for cannabis legalization (coeff. = 0.054, *p* > 0.05), and cannabis use was no longer a significant predictor of support for medical cannabis legalization (coeff. = −0.087, *p* > 0.05). To examine this in more depth, we entered the independent belief variables one at a time. This showed that national and cannabis use differences in support for medical cannabis legalization disappeared when the item about medical effects of cannabis was entered into the model. None of the other belief variables had this same influence on results (results not shown but available upon request).

**Figure 1:**
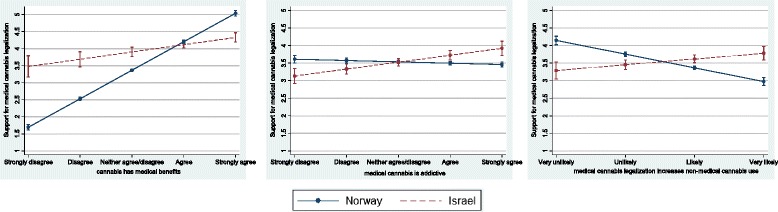
Predicted margins with 95 % CIs for Norway and Israel for support for medical cannabis legalization

To examine policy regime differences in the association between medical cannabis policy support and cannabis-related beliefs, interaction terms and marginal effects were examined. Results presented in model 3 in Table 3 show that all interactions were statistically significant. Figure 1a–c presents the marginal effects for medical cannabis policy support. In graph 1A, the slope for Norway is steeper than the slope for Israel, indicating that the belief that cannabis has medical benefits was more strongly related to medical cannabis policy support in Norway than it was in Israel. Graph 1B shows that in Norway, there is no association between the belief that cannabis is addictive and support for medical cannabis legalization (indicated by overlapping confidence intervals). In contrast, in Israel, a stronger belief that cannabis is addictive was associated with stronger support for medical cannabis legalization. Graph 1C shows that in Norway, the greater concern for spillover effects was associated with less support for medical cannabis legalization. This contrasted with results for Israel where the slope was less steep than for Norway, and the relationship was the opposite; stronger belief in spillover effects was associated with stronger support for medical cannabis legalization.

### Sensitivity analyses

Although we did not make causal claims when analysing and interpreting this cross-sectional data set, it may still be argued that some unobserved variables could influence both the attitudes towards medical cannabis legalization (the dependent variable) and cannabis use in the previous year. These variables could have contaminated the results. While we have no means to fully examine or account for these, a sensitivity analysis was conducted to test the robustness of the reported results, where we excluded last year cannabis users (*n* = 200) and compared the results to those of the original model (model 2). Results from the sensitivity analyses show similar patterns (results not shown but available upon request); the same variables were statistically significant and the coefficients stayed roughly the same. Only secondary education changed from non-significant (*p* = 0.077) to significant (*p* = 0.033) and the coefficient from 0.13 to 0.15.

We also checked the correlation between last year cannabis use and the belief variables in case they had created a collinearity problem. The correlations were generally low, ranging from 0.086 for cannabis use and the spillover belief to 0.252 for cannabis use and the medical benefit belief.

## Discussion

The public, political and research interest in medical cannabis has increased immensely in recent years. A wave of interest into the structural and physiological properties of cannabis and a surge of clinical trials has improved the evidence that cannabis is a promising anti-emetic and anti-pain medication [4, 5, 26]. However, and albeit the emergence of promising evidence, the evidence base supporting its efficacy varies substantially and in general falls short of the standards required for approval of other drugs by the US Food and Drug Administration [10, 27–29].

In addition to this emerging, albeit weak, evidence base, social movements have fought for legal access to medical cannabis. Public pressure to change medical cannabis policies has succeeded in many jurisdictions, yet medical cannabis continues to be controversial because of the potential detrimental public health effects [9, 30–33] and the evolving, yet still weak, evidence base just mentioned. In the midst of these debates and policy changes, and at a time when much is unknown about the future of medical cannabis policies and their potential effects, it is essential to gain a more nuanced understanding of the nature, extent and rationale for public opinion related to medical cannabis policies. Indeed, a better understanding of public opinion can provide a framework for an improved understanding of current and future policy developments.

Our study has examined the relationship between support for medical cannabis legalization and three beliefs commonly underlying medical cannabis debates, namely that (1) cannabis has medical benefits, (2) cannabis is addictive and (3) medical cannabis legalization leads to spillover effects. Given the increased attention to spillover effects in the scientific literature and in the media [9], one may expect that the belief about spillover effects would have been relatively more influential than the other beliefs examined in this study. However, the bivariate analysis shows that the belief that cannabis has medical benefits is most closely related to support for medical cannabis legalization, whereas the belief of the negative effects of medical cannabis use (e.g. addiction and spillover effects) is less important to public support for medical cannabis legalization.

These findings are further strengthened by the multivariate analysis that showed that the national differences in support for medical cannabis legalization (78 % in Israel and 51 % in Norway) were by and large explained by differences in the beliefs that cannabis has medical effects and not by the other belief variables. This strengthens the conclusion that public opinion towards medical cannabis legalization may be mainly driven by deliberations related to the medical effects of cannabis, as opposed to concerns for detrimental public health effects (spillover and addiction effects).

These findings have particular public health and policy implications as they suggest that the recent revival of clinical research and increasing evidence of the medical benefits of medical cannabis [34–37] may be particularly influential in forming and increasing support for medical cannabis legalization. Indeed, this development in basic and clinical research may fuel the public’s belief that cannabis has medical effects, which the current study has shown to be particularly important to public support for medical cannabis legalization.

It has been noted that medical cannabis policy has sometimes been developed without a strong public health agenda [38, 39]. In some jurisdictions, medical cannabis policies have developed in a manner that serves for-profit businesses and large-scale commercialization and marketing of medical cannabis products. One outcome is that a range of consumables, from lollies, chocolate and peanut butter to wine and e-cigarettes containing THC (the main psychoactive compound in the cannabis plant), are widely advertised and available [32]. While the consequences of such trends are yet to be fully understood, studies have found an increase in illicit cannabis use in jurisdictions that have commercialized medical cannabis [40, 41]. Studies have also found an increase in poison centre calls mentioning cannabis in these jurisdictions, although unintentional digestion remains low even after medical cannabis legalization [30, 42, 43]. The observed increase in use and poison centre calls may be caused by greater availability, commercialization and unsafe storage of cannabis in households. However, it is also possible that it is related to increased willingness to report cannabis use and unintentional cannabis exposure in an atmosphere where medical cannabis is legal [9]. Research has also found that more than a quarter of adolescents in a Californian school sample were exposed to advertisement for medical cannabis and that this exposure was associated with greater cannabis use one year later [32]. The relatively weak relation we found between concerns for detrimental public health effects and support for medical cannabis legalization suggests that there may be limited public support for reversing medical cannabis policy in response to the variety of potential unintended consequences.

Another finding of this study is that in Israel, stronger beliefs regarding addiction and spillover effects are related to stronger support for medical cannabis legalization. While this at first seems counterintuitive, it may indicate the acknowledgement of cannabis simply being another prescription drug. Indeed, other pharmaceuticals (and especially those used for chronic pain and cancer pain management) are highly addictive and have spillover effects [44, 45], so negative side effects do not per se imply that such drugs should not be used in medical practice. In Norway, however, where cannabis is less likely to be accepted as a prescription drug, the belief of potential detrimental effects, such as increased recreational use, is associated with weakened support for medical cannabis legalization.

Another possibility is that after years of experience with a state-supported program providing medical cannabis to specific groups of patients, Israelis are aware that such a program is congruent with state policies of prohibition and control over cannabis use for recreational purposes. Thus, it is possible that although addiction and some spillover effect are acknowledged, this does not translate into lack of support for medical cannabis legalization in Israel.

Further, it is worth noting that the belief that cannabis has medical benefits is a much stronger predictor of medical cannabis legalization support in Norway than in Israel. One possible reason for this is that there has been a “legitimacy-conferring process” [46] in Israel whereby medical cannabis legalization has earned legitimacy through expert judgment of authoritative institutions who are responsible for running the medical cannabis program. Such a legitimization process is likely to increase support for medical cannabis legalization in Israel, regardless of the beliefs Israelis hold towards medical cannabis.

Another explanation is that factors not examined in this study could be pertinent to support for medical cannabis policy support in Israel. For instance, direct experience has been shown to be important to belief formation [47]. The fact that Israel has a growing number of medical cannabis patients increases the chances that Israelis will know someone who uses medical cannabis, and this may be an important factor for medical cannabis policy support in Israel which was not captured during our study. Beliefs will also be influenced by knowledge [47]. Since medical cannabis has been on the media agenda in Israel much more than in Norway, it is possible that Israelis are more knowledgeable about the topic and that this is what determines their support for medical cannabis policy. Unfortunately, testing this formally was beyond the scope of our study.

### Limitations

The strength of the study lies in the identical data collection processes and identical questionnaires in two countries with different medical cannabis policies. The study does, however, have limitations that must be considered. Firstly, only two countries were included in the study. More evidence from different jurisdictions regarding beliefs and tolerance towards medical cannabis policies as a function of different policy regimes is desirable. Furthermore, the study was cross-sectional, and thus, no causal inferences could be drawn. As more countries legalize medical cannabis, the opportunity to conduct longitudinal studies that examine the causal relation between public opinion and medical cannabis policy changes will be available. Hopefully, researchers will be able to capitalize on these changes to develop the evidence base further.

Lastly, self-reported data on sensitive topics (such as cannabis use) can be influenced by memory or motivational biases. Contrary to this, research has shown that reports of drug use have high reliability and validity [48, 49]. In particular, web-based reports of cannabis use and related issues have been shown to be valid and reliable [50].

## Conclusion

Medical cannabis is an unconventional and highly contested prescription drug. The increasing and sometimes heated public debates over whether or not medical cannabis should be legalized clearly underline this. This study suggests that public support for medical cannabis legalization is likely to continue to grow. This conclusion is based on the observation that the scientific evidence supporting medical benefits of cannabis seems continuously to grow and also on our finding that the belief in the medical benefits of cannabis is particularly important to public support for medical cannabis legalization. Arguments related to public health and the negative effects of cannabis, on the other hand, have less bearing on public support for medical cannabis legalization. Continued research is needed to investigate how public health considerations can be made more salient in current medical cannabis policy developments, public opinion and deliberations.
